# Theory Integration for Lifestyle Behavior Change in the Digital Age: An Adaptive Decision-Making Framework

**DOI:** 10.2196/17127

**Published:** 2021-04-09

**Authors:** Chao Zhang, Daniël Lakens, Wijnand A IJsselsteijn

**Affiliations:** 1 Human-Technology Interaction Group, Department of Industrial Engineering & Innovation Sciences, Eindhoven University of Technology Eindhoven Netherlands

**Keywords:** behavior change, health behavior, digital health intervention, decision-making, learning, self-control, habits, theoretical framework

## Abstract

Despite the growing popularity of digital health interventions, limitations of traditional behavior change theories and a lack of theory integration hinder theory-driven behavior change applications. In this paper, we aim to review theories relevant to lifestyle behavior change from the broader psychology literature and then integrate these theories into a new theoretical framework called adaptive decision-making to address two specific problems. First, our framework represents lifestyle behaviors at two levels—one of individual daily decisions (action level) and one of larger behavioral episodes (reflection level)—to more closely match the temporal characteristics of lifestyle behaviors and their associated digital data. Second, the framework connects decision-making theories and learning theories to explain how behaviors and cognitive constructs dynamically influence each other, making it a suitable scaffold for building computational models. We map common digital intervention techniques onto the behavioral and cognitive processes in the framework and discuss possible contributions of the framework to both theory development and digital intervention design.

## Introduction

### Background

Digital intervention systems have been considered as promising tools to change people’s unhealthy lifestyle behaviors, such as eating fast food, not exercising, or having suboptimal dental routines [1-4]. In realizing the potential of such systems, many researchers have advocated the role of behavior change theories, especially their translation to digital lifestyle interventions [3,5-8]. Ideally, behavior change theories and digital lifestyle interventions should inform each other. Good theories, when applied appropriately, are generally expected to increase the effectiveness of interventions. They can be used to identify behavioral determinants as intervention targets, translate general behavior change techniques (BCTs) to fine-tuned features in digital systems, and predict intervention outcomes. On the other hand, the vast amount of behavioral data collected by digital systems could potentially contribute to theory evaluation [9-11]. Compared with data from traditional behavioral experiments, digital behavioral data can have larger and more diverse samples, greater ecological validity, and higher temporal resolution.

Despite these expectations, the synergy between theory development and intervention practice is far from ideal [9]. The role of behavior change theories in digital interventions is not as prominent as hoped. Several surveys indicate that the application rate of theories in digital intervention trials and commercial eHealth apps ranges between 19% and 52% [6,12-19], and when theories are applied, only 3-5 classical theories dominate the applications [15,17,19-21]. Moreover, although some reviews and meta-analyses have suggested that applying theories has benefits [22,23], other reviews found no clear evidence [24-26] and questioned the value of applying theories in real-world applications [27]. Finally, as for theory development, data collected by digital systems are commonly used to evaluate the effectiveness of specific interventions but are rarely used to examine predictions derived from theoretical models [25].

One factor contributing to this *theory-intervention* *gap* is the lack of theory integration in the field of behavior change research [28], especially integrations that are tailored to digital lifestyle interventions. Even for a phenomenon as complex as behavior change, the large number of individual theories pertaining to behavior change (83 according to a systematic review [20]) clearly suggests that some integration and unification is probably beneficial for theory development in the field. The sheer number of behavior change theories can be overwhelming for intervention designers who want to grasp the literature and selectively apply theories to their designs. Perhaps the difficulty of orienting oneself with respect to the literature can explain why only a limited set of theories are applied [20]. Many basic theoretical ideas in psychology, despite being highly relevant, are underrepresented in applied research, such as decision-making, reinforcement learning, self-control, and habit formation.

The lack of impact of theories on interventions also raises the question whether the current knowledge about lifestyle behavior change is too limited to be fully useful. Two specific reasons have been proposed to explain why traditional behavior change theories are inadequate in the digital age [6]. First, many prominent traditional theories are static rather than dynamic, in the sense that they provide *snapshot* explanations of what factors determine behavior. Temporal aspects are not taken into consideration. Second, even when time is included in the theories, there is often a mismatch between traditional theories and digital interventions in terms of at what temporal scale behaviors are represented. These two limitations are evident in three of the most applied theories [15,17,19-21]. In the Theory of Planned Behavior (TPB) [29], neither temporal dynamics of the behavioral determinants in the model nor any mechanisms to account for the reciprocal influences of behavior on its determinants are specified. The influential Transtheoretical Model (TTM) includes the temporal aspect of behavior change stages [30]. However, although a healthy-eating app may intervene in its users’ daily dietary choices, the TTM only describes the stages of behavior change in terms of months. If a theory represents behavior at a coarse temporal scale, processes at finer scales are overlooked and time-intensive digital interventions cannot be informed. Other theories, such as the Social Cognitive Theory (SCT) [31], consider more rapid interactions between behavior and behavior determinants (eg, self-efficacy); however, the dynamic interaction is theorized only at a very abstract conceptual level without explicitly modeling the role of time or how the interaction works [32]. Both criticisms also coincide with the recent advocates of paying more attention to temporal aspects in health psychology to improve theories and their translation into practice [32,33].

The two aforementioned points should not be considered as criticism of the original theories. Although these theories have been applied in the context of lifestyle behavior change and digital intervention, they were either not meant to explain lifestyle behaviors initially (eg, TPB) or developed at a time when technologies for continuously monitoring lifestyle behaviors were unavailable (eg, TTM or SCT). To advance theories, it is useful to consider some of the often-overlooked characteristics of lifestyle behaviors through the lens of modern digital technologies. Lifestyle behaviors, such as eating, exercising, or toothbrushing, are performed very frequently, as part of everyday habits and routines, and on each occasion, they are fast decisions that are not extensively deliberated. This type of decisions (eg, choosing what to eat for dinner) may be relatively inconsequential; however, they can form larger behavioral *episodes* (eg, following a diet), which may affect one’s health significantly over time. This characteristic of hierarchical organization sets lifestyle behaviors apart from single-time health behaviors or decisions, such as cancer screening or vaccination. Moreover, unlike single-time decisions, as lifestyle behaviors are repeated frequently, learning and adaptation through repetitions plays a very significant role in lifestyle changes and interventions. This requires the inclusion of temporal dynamics in behavior change theories.

### Objectives

**Figure 1:**
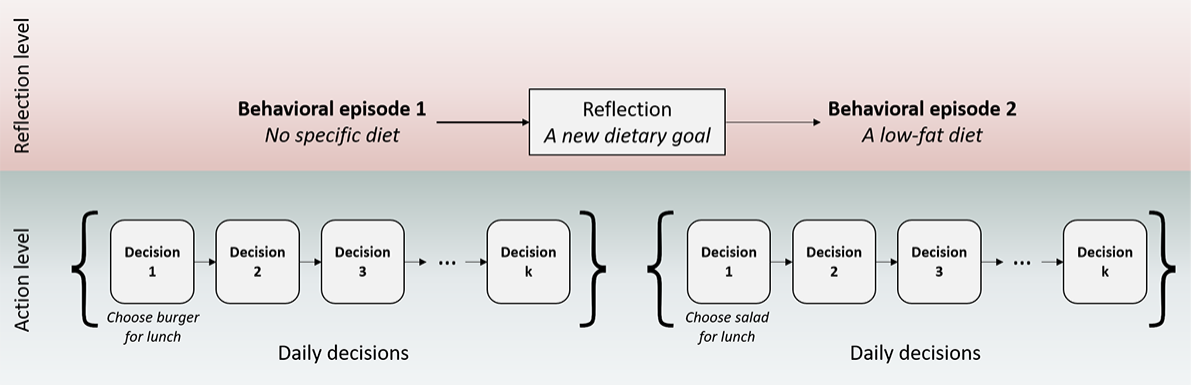
A two-level representation of lifestyle behavior (change).

On the basis of the aforementioned rationale, we propose a new integrative theoretical framework, called *adaptive decision-making*, which specifically focuses on lifestyle behaviors and incorporates temporal dynamics. In doing so, the new framework represents lifestyle behaviors at two temporal levels: a lower level (*action level*) that matches the daily individual decisions and the time-intensive interventions realized by digital systems and a higher level (*reflection level*) that matches the episodes of repeated decisions (Figure 1). In addition, both decision-making processes (how behaviors are determined or decisions are made) and learning processes (how earlier behaviors or decisions influence later ones through cognitive variables) at each level will be included in the framework. The goal is to incorporate both traditional and more recent theoretical ideas about behavior change in a single framework and reinterpret these ideas in light of a fine-grained temporal perspective. We hope this effort will facilitate a more integrated approach for developing more precise theories (eg, computational models) and intelligent intervention systems.

In the remainder of this paper, we first review important theoretical ideas relevant to lifestyle behavior changes from a broad psychology literature. To facilitate theory integration, individual theories are compared in terms of their temporal scales and their emphasis on learning or decision-making. Next, the adaptive decision-making framework is introduced by integrating the relevant but disparate theoretical ideas into a 2-level representation of lifestyle behavior changes presented earlier (Figure 1). Afterward, we relate the framework to intervention practice by mapping common BCTs used in digital systems to the behavioral processes in the framework. The paper concludes with a general discussion on the added value of the framework to behavior change theorists and digital intervention designers.

## Review of Individual Theories Relating to Lifestyle Behavior Change

### Overview

There are two distinct and complementary traditions for explaining human behavior—a learning tradition and a decision-making tradition [34]. The learning tradition, as its name suggests, focuses on the time course of learning a behavior—in particular, the interdependence among behavioral occasions in a sequence rather than the exact determinants of each occasion. In contrast, researchers in the decision-making tradition care more about what factors determine a behavior on specific occasions and what information is processed at such moments but much less on how repeated decisions are interrelated. As both learning and decision-making aspects are crucial for developing a dynamic framework, this review is organized based on the roots of theories in either tradition. After the review, we briefly discuss whether each theory focuses primarily on explaining individual daily decisions (action level) or episodic behavioral processes (reflection level).

### Theories in the Learning Tradition

#### Reinforcement Learning Theory

Reinforcement learning, or learning by outcomes, is a fundamental form of learning discovered in the early years of modern psychology [35] and is still influential in today’s behavioral and brain sciences [36] and artificial intelligence research [37]. Humans and other organisms are theorized to adapt their behaviors through their interactions with changing environments to survive and thrive. If a behavior results in goodness to an organism, the frequency of performing the same behavior increases; conversely, if a bad outcome follows, the behavior will be performed less often in the future. This is summarized as the *law of effect* [38].

Reinforcement theory becomes more complex when one also considers the *law of exercise* [38]. The aforementioned response-outcome learning, or goal-directed learning, is accompanied by stimulus-response learning, also known as a process of habit learning [36]. The distinction between goal-directed learning and habit learning has been demonstrated in instrumental learning experiments where animals or humans are trained to acquire reward-generating responses (eg, pressing a lever to receive food): when a response is overly trained, it persists to be triggered by the corresponding stimulus even when the reward becomes goal irrelevant (eg, when a rodent is satiated) [39]. The recent resurgence of interest in habit formation in social and health psychology also follows the theory of defining habits as mental associations between behaviors and environmental cues [40-42]. When a behavior becomes strongly habitual, goal-related determinants of behavior, such as attitude and intention, cease to influence behavior [43].

#### Control Theory of Self-regulation

The classical reinforcement learning theory focuses on the role of external immediate rewards in controlling behavior but neglects the role of distal behavioral outcomes that may be cognitively represented. Following criticism of this limitation [44], the control theory of self-regulation assumes that people can mentally represent distal outcomes of goals, and the regulation of behavior is generally toward reducing the discrepancies between the goals and people’s current status [45,46]. When a behavior leads to a reduced discrepancy, the reduction itself becomes a reinforcer of the behavior, similar to external rewards. This discrepancy-reduction mechanism is analogous to feedback control systems in engineering, where discrepancies between perceived states and a reference value are constantly monitored to maintain homeostasis.

The control theory also hierarchically represents goals and self-regulation. A 9-level hierarchical control system was proposed by Carver and Scheier [45], in which a behavior output from a higher level serves as the goal reference to the next lower level. For lifestyle behaviors, it is sufficient to consider three levels: long-term goals (eg, improving health), short-term goals (eg, walking 10,000 steps a day), and actions (eg, taking a specific walk). Taking actions leads to the fulfillment of short-term goals, which in turn brings a person closer to the long-term objectives. Self-regulation operates most frequently at the action level (ie, making daily decisions); however, people’s attention can be shifted to higher or lower levels. Downward shifting occurs when lower-level motor control, which is normally highly automated, becomes temporarily impeded during action executions (eg, when learning a new motor skill or when a dysfunctional action has to be inhibited [47]). Upward shifting can be understood as self-reflective moments when a person reconsiders the attainability of a higher-level goal, which is more difficult to predict (Psarra [48]).

#### Social Cognitive Theory

SCT, proposed by Albert Bandura, is one of the most cited and applied theories in behavior change research [20,21]. The theory encompasses three key concepts: *social learning* [49], *self-efficacy* [50], and *proactive control* [31]. First, based on research on children’s learning behaviors [51], *Social Learning Theory* posits that behaviors or attitudes are acquired not only through direct reinforcement but also by observing the behaviors and their corresponding consequences to others [49]. For many health-related behaviors, long-term health consequences are often learned by observing other people’s behavioral outcomes. Second, based on organizational decision-making research [52], it was found that subjective belief in one’s ability to perform a behavior was closely related to actual performance. According to the control theory mentioned earlier, this self-efficacy belief can be understood as a cognitive mechanism that simulates a series of future actions (eg, dinner choices every day) in an extended episode of goal pursuit (eg, adherence to a diet). If the mentally simulated actions fail to bring sufficient progress, a person may decide to abandon the goal pursuit altogether. Third, Bandura [31] was among the earliest scholars to discuss a discrepancy-production process called proactive control, in which a person sets higher goals to further motivate behavior. Thus, it complements the discrepancy-reduction mechanism at the core of the control theory. The idea that goals are susceptible to changes also allows the possibility of adjusting an unattainable goal downward to reduce its discrepancy to the current status. Altogether, the three concepts contribute to extending reinforcement learning and control theory by incorporating flexibilities in complex human behaviors.

### Theories in the Decision-Making Tradition

#### Expected Utility Theory

Across behavioral sciences (eg, psychology and economics), many mathematical models have been developed to describe how people make choices, given a fixed set of alternatives (options, eg, fries or salad) and attributes (eg, healthiness or tastiness). A fundamental theoretical idea behind many models is the expected utility theory. The theory assumes that people integrate multiple attributes of choice alternatives (their potential for satisfying different personal goals) into a unidimensional construct called expected utility and then choose the alternative with the highest utility [53]. Formally, the expected utility is computed as 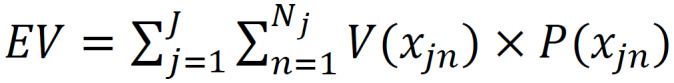, where *V*(*x_jn_*) is the subjective value function for the *n*th possible value of attribute *j*, and *P*(*x_jn_*) is the probabilistic belief that attribute *j* takes that value [54,55]. The equation implies that the expected utility of one choice alternative increases when choosing the alternative is likely to produce certain outcomes (large *P*(*x_jn_*)) and when the outcomes are highly valuable (large *V*(*x_jn_*)). For example, whether people choose salad over fries depends on both their beliefs about their respective benefits for health and their valuations of good health. The theory does not imply that people always consciously follow the equation to compute utilities but rather reflects key neural mechanisms that underlie decision-making [56]. In reality, conscious and deliberative computations are more common for single-time important decisions (eg, comparing different health insurance policies) than for fast daily lifestyle decisions.

#### Sequential Sampling Models

Empirical data from choice experiments have repeatedly shown that people are less rational than those suggested by classical choice models [57]. People are prone to be influenced by information that is seemingly irrelevant, for example, the addition of an inferior choice option [58] or framing of losses versus gains [59]. To account for these anomalies, a sequential sampling approach has been developed to dynamically model the cognitive process of decision-making, such as the *multialternative decision field theory* [60] and the *associative accumulation model* [57]. The new models share the idea that preferences for different choice alternatives are accumulated over time (eg, a few seconds) and the choice that is made is the choice whose preference signal is first to exceed a decision threshold. At each time step, the preference signals of choice alternatives fluctuate according to a process of utility comparison based on one [57] or multiple attributes (as in drift diffusion models [61]). The stochastic property of sequential sampling models enables them to explain the sensitivity of choices to subtle changes in choice sets and to predict decision time [56]. Finally, sequential sampling models suggest a mechanism for habitual choices, where repeatedly choosing an alternative may shift its starting position of preference accumulation toward a decision threshold at the baseline ([60], Zhang et al, unpublished data, 2021).

#### Reasoned Action Approach

Influenced by the expected utility theory [62] but with a strong focus on application, the reasoned action approach [63] has produced some of the most applied theories in behavior change research, such as the TPB [29,64] and the Health Belief Model [65]. From a decision-making perspective, this approach categorizes attributes in certain choice situations into a smaller set of behavioral determinants that are generalizable to a wide range of behaviors and measurable by self-report. For example, in the TPB, regardless of the specific alternatives and attributes considered, factors affecting choices are categorized into three determinants, namely attitude, social norm, and perceived behavioral control [64]. When a specific behavior is considered (eg, dinner choice), attitude toward a choice alternative is further determined by many attributes [29], such as taste, nutrition, and price, whereas social norms are influenced by the perceived social consequences of choosing an alternative (eg, presenting oneself to be environmentally friendly). Perceived behavioral control, similar to self-efficacy, measures one’s confidence in maintaining certain choices in the future. TPB was explicitly considered by Prochaska and DiClemente [29] as a model for behavioral prediction rather than for explaining the processes underlying overt behaviors or decisions or how such processes can be influenced.

The reasoned action approach also makes a strong assumption on the intentionality of behavior [66]. For example, behavioral intention is a prerequisite for actual behavior in the TPB [64]. Thus, this approach considers behaviors as *planned* or *intended*, resulting from careful deliberations on the pros and cons of certain behaviors. Such a theoretical position is reasonable because the reasoned action approach was developed to mainly deal with single-time decisions or the planning of behavioral episodes, rather than *small* daily decisions. When applied to lifestyle behaviors, this approach relies on aggregated behavior representations over a substantive period [67].

#### Dual-Processing Models

A recurrent idea in psychology is that humans possess two distinct modes or systems for processing information and making decisions. Although different dual-system models use different terminologies [68], it is widely accepted that one system is fast, impulsive, and largely automatic, and the other system is slow, reflective, and deliberate [69].

The Reflective-Impulsive Model [70] is a representative of this approach, and it has been explicitly applied to health-related behaviors [71,72]. The reflective system hosts various higher-order mental operations that rely on controlled processes and symbolic representations, including deliberate judgments, planning for goal pursuit, and inhibition of prepotent responses. In contrast, the impulsive system operates fast on associative clusters in long-term memory that group stimuli, affective states, and behavioral responses together. At the moment of a specific decision, the success of self-control depends on the relative ability of the processes in the two systems to activate the corresponding behavioral schemas. Several boundary conditions have been proposed to moderate the relative strengths of the two systems [72]. For example, people are believed to behave more impulsively when their behaviors are highly habitual, when their cognitive loads are high, and when their moods are positive.

### Temporal Scales Used in the Aforementioned Theories

**Figure 2:**
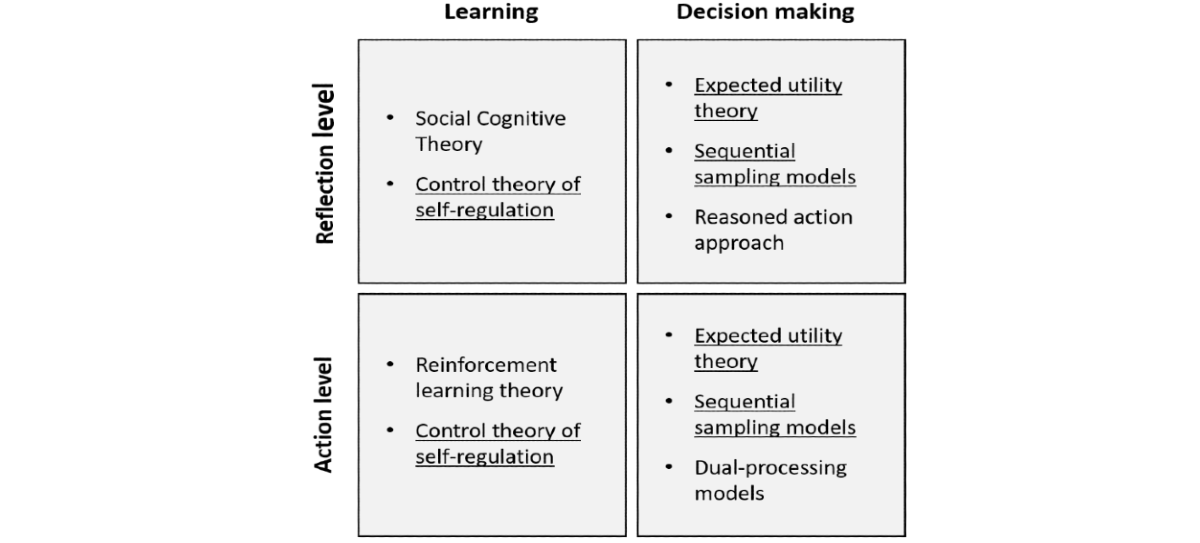
Categorization of reviewed theories based on their theoretical traditions and temporal scales (theories that apply to both temporal scales are underlined).

Figure 2 summarizes the learning and decision-making theories based on the temporal scales of behavior representation. A similar distinction was made by Karoly [66], where theories at the action level were called *online* theories and theories at the reflection level were called *offline* theories.

In the learning tradition, the reinforcement learning theory clearly represents behavior at the action level, as the outcome of each specific action or decision is modeled to have concrete impacts on the frequency of repeating the same action in the future. Reinforcement learning experiments also involve repeated trials within a relatively short period (eg, a few hours). The control theory of self-regulation, because of its hierarchical structure, covers both behavioral processes at the reflection level and the action level. SCT and its processes of self-efficacy and proactive control apply mainly to behaviors at the reflection level. Although the two processes may have counterparts at a lower level, as in the control theory, Bandura’s [31] focus was clearly on voluntary and deliberative human behaviors.

In the decision-making tradition, mathematical models as part of the expected utility theory and the sequential sampling approach can be equally applied to decisions at both temporal scales, as long as decisions with clearly defined choice sets are considered. As discussed earlier, theories in the reasoned action approach deal mainly with decisions at the reflection level because of its assumption of intentionality. In contrast, dual-process models are mainly intended to account for *small* daily decisions, for which both reflective and impulsive processes play a role. There is a difference between *reflective* used in dual-processing theories and what we mean by *reflection level*, which will become clear after theory integration.

## Theory Integration: An Adaptive Decision-Making Framework

### Overview

To reiterate, our goal of theory integration is to develop a unified framework that identifies and connects all relevant decision-making and learning processes at both the action and reflection levels of lifestyle behavior change. Most processes in the framework came directly from the theories reviewed earlier, but efforts were made to unify different terminologies from different theories to form a coherent framework and to tailor the framework to lifestyle behaviors. Taking dietary behavior as a primary example, the framework should explain not only how daily meal choices are made and how each decision outcome influences future choices but also how the goal of adhering to a specific diet is made and how such goals are evaluated. The following sections introduce the adaptive decision-making framework in four parts: action-level decision-making, action-level learning, reflection-level decision-making, and reflection-level adaptation.

### Action-Level Decision-Making: Daily Meal Choices

**Figure 3:**
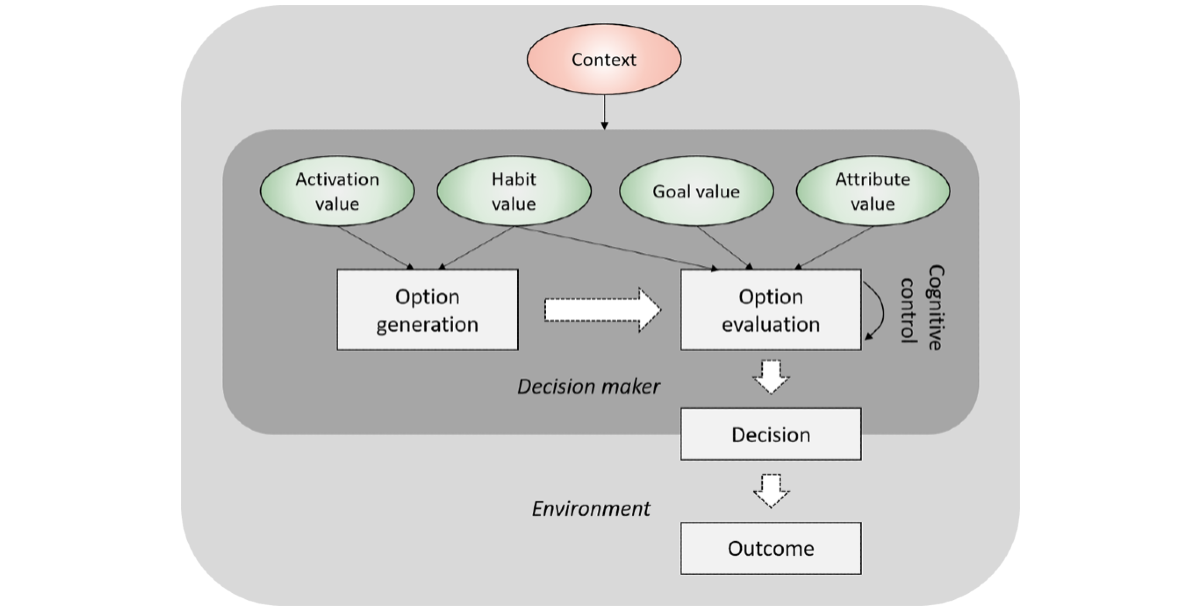
A two-step model of daily lifestyle decision making.

Daily lifestyle decisions, such as daily meal choices, can be modeled as a two-step process—*option generation* and *option evaluation* (Figure 3). The framework assumes that when choosing a meal, different meal options must be generated or recalled by a decision maker first, before evaluations of a few options can be made to inform a final choice [73,74]. The notion of option generation has not been examined in any of the decision-making theories reviewed, probably because most of the theories are based on laboratory choice experiments, where options are simply provided by the experimenters. For lifestyle behaviors in daily environments, how choice alternatives are generated is an important question.

In general, behavioral options can be generated using three different methods. First, if an option is habitual, it will be activated when the associated cues are encountered, such as location and time (eg, lunch at the office) or a combination of contextual cues (eg, a busy Wednesday evening). Second, options may be remembered at the right moments because people intentionally try to maintain them in their prospective memory (ie, not to forget to do something in the future [74,75]). This usually happens when there is a salient goal guiding daily decisions, such as the goal of adhering to a low-carb diet. People may also intentionally associate important options with external cues so that encountering cues is likely to trigger the options [76]. Third, options can be triggered by direct external suggestions at the decision moments, for example, a coaching message from a mobile health app that recommends healthy foods [73]. Through these means, behavioral options that are sufficiently activated (eg, by passing an activation threshold) will be evaluated.

Option evaluation can be modeled as a process of comparing several options and then choosing the one with the highest goal-satisfying value. The exact computation of utilities can follow either classical expected utility models or more dynamic sequential sampling models. Here, it is sufficient to identify three main cognitive variables in the evaluation process. First, when multiple personal goals are relevant for a daily decision, these goals can be regarded as more or less important by a decision maker, thus entailing higher or lower *goal values*. For example, between the goals of living a healthy life and enjoying delicious food, a person who regards the former goal as more valuable would be more likely to choose food options for meals that satisfy their health goals.

Second, for each personal goal, a behavioral option has its *perceived attribute value* relating to that goal, which determines the total utility of the option. These attribute values are subjective beliefs held by people about the causal relationships or contingencies between choosing certain behavioral options and the realizations of personal goals. Although goal values are relatively more stable within-person, attribute values are more context-dependent and prone to changes through learning and experience. For example, the perceived taste of a particular meal option may depend on a person’s momentary appetite, and it may change over time through repeated tasting of the food (ie, habituation [77]).

There is a particular challenge for making healthy decisions, as usually two distinct types of attributes are considered: an immediate hedonic aspect such as tastiness and a long-term consideration of health consequences. From a decision-making perspective, this challenge is essentially a problem of self-control [78]. According to the idea of temporal discounting in decision-making theories [79,80], as any reward from potential health improvements is delayed in time when compared with the immediate hedonic aspects, the value of the attribute healthiness is discounted before it is integrated in option evaluation [81,82]. Another reason why health aspects are often weighted less than hedonic aspects in actual decisions is that the former are more abstract concepts so they might be more difficult or take longer to be processed [83-86]. Finally, from a dual-processing perspective (eg, [54]), dietary self-control may sometimes succeed because people can voluntarily exert top-down cognitive control on the option evaluation process, especially if a momentary preference for a meal option conflicts strongly with a diet goal. It has been shown experimentally that cognitive control may either modulate the valuation process to be more in favor of healthiness rather than tastiness [87] or filter people’s attention away from hedonic attributes in the early stage of option evaluation [88]. Effective top-down control depends on many contextual variables, such as motivation [89], mental fatigue [90], stress level [91], and daily affective states [92].

Third, *habit values* or habit strengths, which represent the history of choosing certain behavioral options, may influence the evaluation of options. As mentioned earlier, learning experiments have shown convincingly that even when two options are provided to decision makers, habitual options are more likely to be chosen than nonhabitual options [36,39]. In sequential sampling models, the influence of habits on the dynamic process of option evaluation can be understood as positively biasing the baseline preferences for habitual options ([60], Zhang et al, unpublished data, 2021). As an intuitive example, if someone often chose fast food in the past, fast food is by default more favorable than other options when no additional deliberations are made.

### Action-Level Learning and Adaptation: Developments of Eating Habits

**Figure 4:**
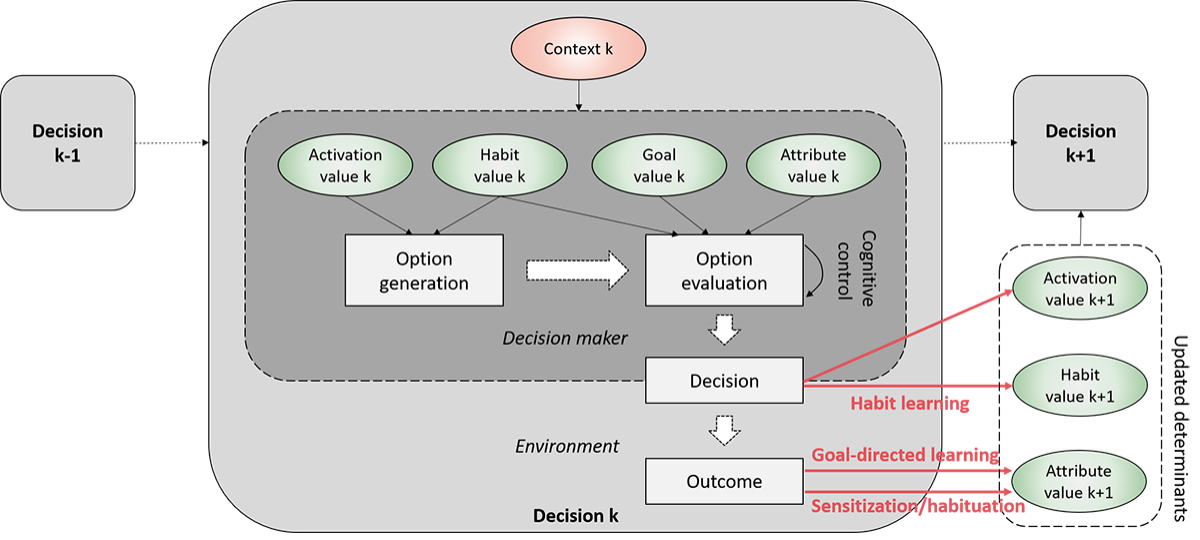
Action-level learning processes added to the decision-making model.

Action-level learning processes can be added to the framework by integrating the ideas of goal-directed learning and habit learning from the reinforcement learning theory to the two-step decision-making model proposed earlier (Figure 4). First, feedback from decision outcomes to perceived attribute values represents goal-directed learning. For example, when a new canteen is opened at a workplace, employees may have initial but very uncertain beliefs about the tastes and calories of different lunch options; however, after a few weeks of trying them out, they gradually form more accurate perceptions about the options. Computationally, the updates of perceived attribute values can be done through model-based and model-free reinforcement learning algorithms (eg, temporal difference learning [37]) or Bayesian belief update [93]. For health-related attributes, because concrete decision outcomes are infrequent except for extreme cases (eg, food poisoning), it is less clear how direct learning from experience works, if it is possible at all (Gershman and Daw [94]). People’s beliefs about the health consequences of different foods are more susceptible to social learning and education.

Second, there is direct feedback from decisions to habit values, as in the process of habit formation or habit learning. Although daily lunch decisions in a new canteen are driven primarily by goal-related attribute values, through repeated decisions, mental associations between frequently chosen food options and environmental cues (eg, the physical setting of the canteen or lunchtime) are gradually strengthened. These associations, as habit values, influence future decisions through both the option generation and option evaluation processes, as discussed earlier. The exact mechanism of habit learning is beyond the scope of this paper; however, it has been modeled in the literature [48,72,95,96].

Third, there is also a direct link between decisions and the activation values of options, which has been discussed much less in the learning literature. When a decision is made and the corresponding behavior is executed, the behavior execution increases the activation level of the behavioral option in memory, although such an increase has been shown to be very small empirically [72]. As discussed earlier, the dynamics of activation values are primarily memory processes and are mostly affected by physical and social stimuli in the environment.

Fourth, with repeated daily actions and exposure to the sensory outcomes of the actions, people’s neurological responses to the same sensory stimuli, depending on the parameters, may either intensify (sensitization) or fade away (habituation) [97]. This action-level adaptation is especially important for eating behavior, for which the exact same food becomes less palatable over time and accordingly its consumption will decrease [77]. This explains why the large variety in the modern food industry is considered a contributor to obesity [98]. In our framework, sensitization or habituation can be represented as an additional mechanism of sensory feedback from decision outcome to attribute value, in addition to the more cognitive process of goal-directed learning.

### Reflection-Level Decision-Making: Dietary Goal Setting

Action-level decision-making and learning processes cover a substantial part of what people do in their daily lives. They depict lifestyle behaviors as repeated decisions without much purpose. However, people also have moments when they reflect on their health status, contemplate possible improvements, and make concrete plans. According to the control theory [45], these reflective processes require short-term goals that bridge people’s abstract long-term goals (ie, what they pursue in their lives) and action-level daily decisions. Short-term goal setting can also be understood as a process of decision-making, albeit at the reflection level rather than the action level. The decisions made are commitments to goals that guide future daily decisions rather than overt behaviors that trigger motor programs.

Therefore, the two-step model of action-level decisions also applies to the setting of short-term goals. In selecting a dietary plan, for example, people first search for diet options that serve their long-term goals and then evaluate the options on relevant attributes, such as taste, ease of preparation, and expenses. At the reflection level, these attributes are often categorized into a few determinants, such as attitude, social norm, and perceived behavior control [29]. Nonetheless, goal setting differs from action-level decisions in some respects. First, because goal setting is less frequent than daily decisions, strong habits are unlikely to be formed to influence decision-making processes. Potential biases by habits are further reduced because people are more careful and take more time to generate goal options and evaluate them. Second, because people set goals for an extensive period of time in the future, they may form a more abstract mental construal [99], which is detached from direct sensory information and visceral attributes, such as effort and tastiness. Thus, the self-control problem for daily lifestyle decisions is less prevalent in goal setting. Third, self-efficacy plays an important role in goal setting [50]. People may carefully consider the feasibility of different diet goals by mentally simulating a series of daily dietary choices in the future.

#### Motivating Functions of Short-Term Goals

**Figure 5:**
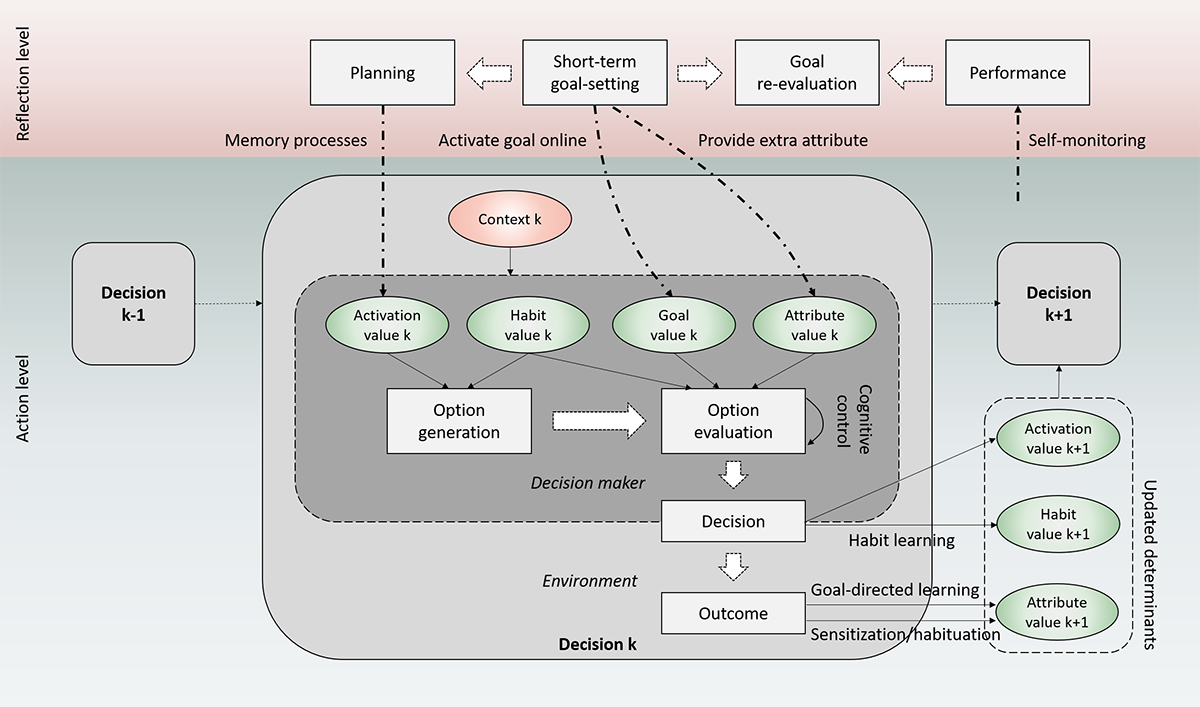
A full representation of the adaptive decision-making framework (reflection-level processes and interactions between the 2 levels added to the previous decision-making model).

When short-term goals are generated, they can influence daily lifestyle decisions through both option generation and option evaluation (Figure 5). First, setting up a short-term goal can increase the activation values of desirable behavioral options through a process termed *planning*. Planning can be done through two mechanisms discussed earlier: an effortful prospective memory process (eg, rehearsing eating salads [75]) or an *implementation intention* process, that is, mentally associating a behavioral option with certain environmental cues (eg, eating an apple when watching television [76]). Second, compared with long-term goals, short-term goals are more concrete; therefore, complying with these goals brings immediate satisfaction [100]. Goal-compliance satisfaction functions as an additional attribute that competes with other hedonic attributes in the option evaluation.

### Reflection-Level Learning and Adaptation: Self-monitoring and Re-evaluation of Dietary Goals

Short-term goals must be re-evaluated in reflection moments periodically to change the goals that are too difficult, too easy, or no longer relevant. Such reflection-level adaptation processes are well described by the control theory [45] and SCT [31]. Goal re-evaluation first requires inputs from action-level processes through *self-monitoring*. Through repeated daily dietary choices, past choices and their outcomes are stored in episodic memory and are later retrieved and integrated into a mental representation of overall past performance (Figure 5). Next, discrepancy between a goal reference (eg, a dietary plan) and a performance representation is computed and used to inform the reflection-level adaptation. Depending on the size of discrepancies and other contextual factors, people may motivate themselves further to make healthy daily dietary choices to reduce the goal performance discrepancies. However, when discrepancies are deemed too large, people may instead lower their goal standards (eg, be less strict on calorie intake) or abandon their goals altogether (eg, give up a diet). Instead, when performance matches or even exceeds current goal standards, they may proactively adjust their goal standards upward to further improve their health [31].

## Mapping Digital Intervention Techniques to the Framework

We define *digital intervention techniques* as BCTs or behavior change methods that target lifestyle behaviors and are implemented in digital systems (eg, web, mobile, or wearable systems). BCTs, in turn, are generally defined as the active ingredients of interventions that can influence behaviors in desirable ways [34,101,102]. Mapping BCTs to theoretical constructs is an important exercise when trying to evaluate and enhance the effectiveness of the techniques; such mappings have been carried out previously [102,103]. Focusing on their implementations in digital intervention systems, our mapping exercise is the first to connect BCTs to theoretical constructs in a single unified framework. Specifically, we categorize and interpret digital BCTs according to targeted behavioral processes and cognitive variables in the framework. The strengths and limitations of some techniques are discussed based on the implications of the framework.

### Digital Intervention Techniques Targeting Action-Level Decision-Making

As digital systems are prevalent in people’s daily lives, they are well positioned to influence people’s daily lifestyle decisions at the time of the decision. The ability to target action-level decisions is considered by many as a promising direction for digital lifestyle interventions, as reflected in research on *ecological momentary interventions* [1] and *just-in-time adaptive interventions* (JITAIs) [104-108]. According to our framework, there are many different ways in which digital intervention systems can influence online decision-making processes, depending on whether the techniques target option generation or option evaluation and which cognitive variables are targeted; four main categories can be distinguished.

#### Option-Based Techniques

Option-based techniques make certain desirable behavioral options salient but leave the evaluation of options to users themselves. When a desirable behavior is obvious but may not be constantly salient to users, digital systems can simply prompt users to actively make decisions to engage in that behavior, for example, to take breaks when overly sedentary behaviors are detected by the system [109,110]. Otherwise, it might be possible to provide users with new options that are better than those known by users themselves [73]. Finding such *attractive* options relies on a system’s sensor network and smart algorithms, which potentially make it more knowledgeable than its users in a given behavioral domain or context. For example, a smart system was developed to recommend new commuting routes to users in situ to increase physical activities, based on automatic detection of users’ habitual routes and Google Maps data [111].

#### Attribute-Based Techniques

Attribute-based techniques aim to change users’ beliefs about the attribute values of options by providing health-related knowledge or facts. They are referred to as *providing information about behavior-health links* or *providing information about consequences* in the taxonomy of BCTs [101]. Given the common assumption that humans are rational decision makers, providing information about attribute values is a logical approach to behavior change and has been used extensively in traditional health education campaigns. However, attribute-based techniques alone do not guarantee successful behavior change, as attribute value is only one of many factors that influence decision-making. It is also questionable whether digital systems are better tools for providing such information when compared with human experts (eg, lifestyle coaches). Nonetheless, information about attribute values can be provided to justify the recommendations of behavioral options whenever appropriate in digital systems (eg, calorie information for different meal choices).

#### Goal-Based Techniques

As goal values modulate attribute values in option evaluation, activating health-related goals in the decision moments provides yet another type of intervention technique. When implemented in digital systems, they link the suggestions of concrete behavioral options with the reminder of associated short-term or long-term goals. For example, when a mobile app prompts a user to take a lunch walk, the user’s goal of walking 10,000 steps a day (and the achieved steps) can be presented along with the option of taking a lunch walk.

#### Structure-Based Techniques

Structure-based techniques differ from previous types because they neither change the availability of options nor alter users’ existing beliefs about attribute values. As they require less processing effort and are less susceptible to reactance from users than other techniques, structure-based techniques have attracted significant research interest [112,113], usually under the name of *nudging* or *choice architecture* [114,115]. For example, people can become more likely to choose the desirable options when they are presented as default options [116] or an additional option is introduced to change their perceptions of choice sets (context effects [57]). Lee et al [117] adopted the default technique to promote healthy snacking in an online environment by making healthier options the default choices. Zhang et al [118] built on a context effect called the *compromise effect* to promote physical exercise at work using a mobile app. Intensive exercise options were added to make moderate exercise options appear more achievable and thus more attractive [118].

A challenge for all JITAIs is that most lifestyle behaviors are *physical* rather than *digital* in nature. When making decisions about snacking, exercising, or toothbrushing, people do not naturally come to digital applications. In contrast, in e-commerce, for example, people are accustomed to shopping online; therefore, e-commerce sites such as Amazon never miss the opportunity to influence consumers in their decision moments. To influence lifestyle decisions at critical moments, interfaces between the information in digital systems and people’s spontaneous behaviors in the physical world need to be created. Current approaches include predicting users’ spontaneous decision-making moments using sensor networks (eg, predicting *about-to-eat* moments [119]) and initiating decisions when interventions are predicted by the system to be most valuable (eg, predicting stressful moments [120]). This challenge will continue to stimulate new intelligent digital solutions and at the same time debates on the associated ethical implications [121].

### Digital Intervention Techniques Targeting Action-Level Learning

Digital intervention techniques targeting action-level learning processes operate in between rather than at decision moments. The goal is to support either goal-directed learning or the formation of healthy habits. If these techniques are effective, the cognitive variables that influence decision-making will be in a health-promoting state, so that users are expected to maintain the learned healthy behaviors without continuous intervention by digital systems.

A main challenge for lifestyle behavior change is learning the causal relationships between one’s behaviors and health consequences, as these consequences are usually delayed. As discussed, researchers have speculated on the role of episodic memory in tracking internal and external events to support this type of learning [94]. In this regard, the self-tracking function of many digital systems can support learning by externalizing the user’s memory systems [98]. Behavioral and contextual data can be objectively recorded and reviewed later by users when consequential health events occur. As self-tracking studies mostly focus on the effectiveness of the technology as a whole, evidence regarding its specific role in supporting goal-directed learning is lacking [122]. Some interview data indicated that users of self-tracking systems believed that they acquired knowledge about behavior-health links through self-tracking technology [123,124].

Instead of directly supporting the learning of health consequences, another popular approach is to provide extra rewards that may reinforce desirable behaviors. In the so-called gamification systems, the most common extra rewards are virtual rewards, such as points, badges, or rankings in leaderboards [125-127]. These virtual rewards are expected to steer users to healthy behavioral options by competing with the inherent hedonic values of many unhealthy behaviors.

Despite its popularity, the effectiveness of virtual rewards in their simplest forms is questionable, as empirical studies found no positive effects in several health domains, such as physical activity [128] and sexual protection behavior [129]. Moreover, users in one study perceived such virtual rewards implemented in an exercise-promoting app as *not motivating* or even *unnecessary* [130]. Our framework implies that the problem with virtual rewards is not in the learning of the contingencies between behavioral responses and rewards but in the corresponding goal values of these rewards: the goal values of virtual rewards are often low, when compared with other hedonic attributes, such as tastiness and reduced effort. Future research on gamification should focus on making virtual rewards more goal relevant and meaningful [131], for example, by embedding them as a game mechanic that users care about [132], using tangible rather than intangible rewards [133,134], or making the rewards socially meaningful [135,136].

Another technique in this category is habit formation support, usually by reminding users about a new and desirable behavioral option. This is especially valuable at the beginning of habit formation when new options are not always remembered by users themselves. Unlike the technique of suggesting options at decision moments, reminders that support habit formation are sent offline and according to time-based schedule (eg, once every morning). They do not persuade users to act immediately but to increase the activation values of certain options so that they are more likely to be generated when decision moments arrive. Reminders have been widely used and have been shown to be effective in domains where forgetting is the main obstacle for behavior change [137,138]. More research is warranted to understand its value in changing more complex lifestyle behaviors when the activation value is one of several cognitive variables.

### Digital Intervention Techniques Targeting Reflection-Level Decision-Making

Setting up a short-term goal as a reflection-level decision-making process is often the starting point of self-directed behavior change [139]. Without external interventions, goal setting can be triggered under specific conditions, for example, when someone has learned new health-related knowledge (eg, become aware of the risk of smoking) or has experienced a sudden change in their health status (eg, being diagnosed with diabetes). Thus, a straightforward intervention technique is to proactively prompt users to set up new goals to improve their lifestyles. In many digital systems, following a goal-setting prompt, a user can choose a goal and then record it in the system, which allows the system to remind the user of the goal when needed.

As goal setting is a decision-making process, most techniques discussed in the section on targeting action-level decision-making also apply to goal setting, including option-, attribute-, and structure-based techniques. As a particularly promising direction, digital systems may use their data-gathering power and artificial intelligence to recommend novel and attractive options for short-term goals [73]. To address the subtlety and complexity of goal setting in the health domain, the systems need to personalize options based on users’ abilities [140] and based on their unique life experiences and personal context [141-144]. In the future, the difficult task of setting up challenging, motivating, yet realistic goals may indeed be transferred from people to intelligent intervention systems, at least in part.

After the goal-setting step, digital systems can go further to support the planning phase that connects short-term goals to daily decisions in the future. A simple technique is to prompt users to make concrete plans in the system, for example, by adding activities to a calendar. Data provided by users allow digital systems to check user adherence and send reminders when necessary. In addition to this time-based planning technique, digital systems may encourage users to use the event-based planning technique of implementation intention [76]. Implementation intention has been shown to be effective in the health domain [145,146], and it has also been implemented in digital interventions where no human instructions are required [147,148]. A recent system even uses sensor data to automatically generate *if-then* rules adapted to the living contexts of individual users [149].

### Digital Intervention Techniques Targeting Reflection-Level Adaptation

At the level of reflection-based adaptation, providing behavioral feedback to users to support self-monitoring is the most commonly used BCT in digital systems [19,150-153]. Technically, with the development of increasingly powerful sensors, digital systems are able to track lifestyle behaviors and related variables more accurately and in greater detail than people’s own memories. Moreover, these systems can transform the rich raw data into numerical or visual information (eg, weekly summary of step count) to facilitate better comparison with short-term goal references [122].

Although self-monitoring as a general BCT has been identified as effective [154], the evaluation of this technique in digital systems has yielded mixed results [153,155]. The evaluation is also impeded by the lack of high-quality studies and a lack of focus on self-monitoring per se [122]. It is evident that the abundance of self-tracking devices has not solved the problem of lifestyle behavior changes. From an evolutionary perspective, as people’s natural self-monitoring function has existed long before the existence of digital systems and quantitative data, it is not self-evident that technology-enhanced information would lead to better functioning. A recent study indicates that some self-tracking users may have an exaggerated focus on numeric feedback as the replacement of bodily experience as feedback, potentially leading to negative consequences such as rumination [156]. The bottom line is that even if technology-enhanced self-monitoring is beneficial to some extent, our framework implies that it is only one step in reflection-level adaptation. Future research should investigate how digital systems can also support reflective processes that immediately follow self-monitoring, including the comparison between goal references and monitored performance and the adjustments of goals and behaviors.

## Discussion

### Overview

Understanding and changing lifestyle behaviors in the digital age require a theoretical perspective that combines decision-making and learning and a representation of behavior at the level of both daily decisions and episodic reflections. These two requirements have guided our review of individual theories and their integration, and the outcome is temporally fine-grained, dynamic, and process-oriented theoretical framework of lifestyle behavior change. Through a mapping exercise, we also linked common digital intervention techniques to behavioral processes and cognitive constructs in the framework.

### Theoretical Contributions and Comparisons With Previous Integration Works

The primary objective of developing an adaptive decision-making framework is to address the mismatch between theory and digital intervention in terms of temporal granularity [6]. This was done by considering lifestyle behaviors at two different timescales, one representing the individual daily decisions or actions and one grouping the repeated daily decisions into a larger episode and incorporating self-regulatory processes. This 2-level representation adds value over previous integration attempts that were based on the stage model of change [30], such as the computerized behavior intervention (COMBI) model [157,158] and the i-Change Model [159]. Although the COMBI and i-Change Model postulate a more general process of behavior change (eg, through contemplation, preparation, action, and maintenance), our framework allows one to zoom to a finer level of granularity to explain how repeated daily actions, with the help of reflection-level regulatory processes, lead to maintenance. With time-intensive behavioral monitoring becoming more accessible, our framework compliments earlier work by motivating future intelligent systems that update users’ behavioral and cognitive states after every daily lifestyle decision (eg, computing self-efficacy [160]). In our own work, we proposed a system that computes users’ habit strengths of toothbrushing based on sensor-measured behaviors [161].

There are previous frameworks that are more similar to our adaptive decision-making framework when it comes to behavior representation. Both PRIME (Plan, Responses, Impulses, Motives, and Evaluations) theory [162] and Temporal Self-Regulation Theory [163] model behavior change as a continuous process rather than a series of discrete stages. However, our framework explicitly distinguishes between the two distinct levels of lifestyle behaviors and the different timescales involved. Note that similar to many other dichotomies used in psychology (eg, impulsive vs reflective, unconscious vs conscious), although our 2-level dichotomy is a simplification of a potential continuum of processes, it is still useful for developing new theories, empirical research, and applications. The conceptual distinction is important because it enables our framework to represent lifestyle behaviors at the same temporal granularity with time-intensive behavioral data while incorporating cognitive processes that are detached from daily decisions (eg, goal setting or planning).

A second limitation in the current literature is the lack of dynamic processes in traditional behavior change theories [6]. By integrating theories from both learning and decision-making tradition, our framework depicts a dynamic bidirectional relationship between behaviors and cognitive variables that influence behaviors. The framework complements previous frameworks that focused exclusively on learning processes, such as the framework of evolutionary learning processes [97] and Action Change Theory [164]. More broadly, we believe that the need to capture the complexities of lifestyle behaviors for designing better digital interventions provides a strong and timely motivation to integrate decision-making and learning theories in basic psychological research [165].

Furthermore, this study may stimulate some rethinking about the popular dual-processing models, in which health behaviors are assumed to be driven by two distinct forces, one reflective and one impulsive [72]. In our view, such dichotomous categorization of diverse processes and constructs might be too coarse for a full understanding of the dynamic lifestyle behavior change process. The adaptive decision-making framework suggests several dualities. First, there is a contrast between long-term health benefits and immediate hedonic rewards in option evaluation, where effortful cognitive control is required to battle impulses. Second, goal-directed evaluation based on attributes (both long-term and short-term) competes with the influences of habits. This has been discussed extensively in the learning literature as a dual action control by goals and habits [166]. Third, the faster processes at the action level can certainly be contrasted with the more deliberative processes at the reflection level, which also operate on a much slower timescale. Note that this distinction between action and reflection levels certainly reminisces the classical distinction between motivation and volition [167] and the related Rubicon model of action phases [168].

Finally, the process-oriented nature of our framework makes it an ideal scaffold for developing new dynamic computational models envisioned by many researchers [6,104,169,170]. The processes and mechanisms in the framework are described at a level of specificity that allows their transformation into computational models by introducing additional assumptions and formal algorithms. For example, key cognitive variables are defined for option evaluation, but the exact computational process of how these variables are integrated to produce decisions is left open to different modeling possibilities [44,171]. Similarly, although the framework acknowledges the joint influence of habit and goal-directed control, the exact arbitration between the two is subjected to different computational accounts [96,172,173].

### Added Value to the Synergy Between Theory and Digital Intervention

The adaptive decision-making framework was developed with the aim of narrowing the gap between behavior change theories and digital intervention applications. As a first step, the framework provides a summary of the main theoretical ideas in psychology relevant to applied behavior change research. As such, it can be used as a reference if practitioners want to read more about specific theories and computational models. The integration of both traditional and more cutting-edge theories in the framework should help practitioners to see familiar theories (eg, self-efficacy and goal setting) in a new light and hopefully motivate them to experiment with new ideas (eg, habit formation or sequential sampling models of decision-making). What is especially promising is the prospect of designing intelligent close-loop intervention systems based on computational models informed by our framework. In the current best practice, behavior change determinants and corresponding BCTs are identified and then carefully translated into functions in digital applications; however, their effects on behavior are simply speculated (open loop). By relying on computational models, intelligent systems can monitor behaviors that are targeted and use behavioral data to update the cognitive variables of users to deliver JITAIs for each individual user [107,174]. Examples of such close-loop systems can be found in recent works by other researchers [160] and ourselves [161].

Moreover, our framework’s emphasis on behavioral processes and their corresponding digital intervention techniques should contribute to the identification, implementation, and evaluation of intervention techniques. First, similar to an intervention mapping approach [102], our framework makes a clear distinction between people’s behavioral processes and the techniques that may influence these processes, which is not always made in other coding taxonomies. For example, *habit formation* has been considered a BCT [101]; however, it is essentially a behavioral process that also operates without interventions and is driven by multiple lower-level processes. It is more informative and actionable for system designers if they are informed about the specific processes underlying habit formation and how they can be changed rather than simply implementing a technique called habit formation. Second, by mapping digital intervention techniques to behavioral processes in our framework, it should become clear that a technically well-defined function often targets multiple distinct behavior processes. For example, self-tracking may increase users’ knowledge about behavior-health links but may also support self-monitoring [122]. We argue that evaluation research (eg, review and meta-analysis) should focus more on the effects that specific intervention techniques have on individual processes rather than the effectiveness of broadly defined categories of technologies (eg, *feedback system* [155]), to gain a better understanding of how and why certain intervention techniques work. Third, when combining multiple intervention techniques within a single digital system, our framework can inform designers about whether the techniques target complimentary processes and constructs or the same process and construct. In the latter case, the combination of techniques as a package may not necessarily be more effective than its components, and more careful analysis is needed. For example, as implementation intention and *just-in-time* reminder both increase the activation values of desirable options, it is questionable whether combining them would yield better results (Luszczynska et al [147]).

The adaptive decision-making framework may also help to encourage the use of digital lifestyle intervention systems to advance basic human behavior sciences. We have emphasized in this paper the unique opportunities of *bringing psychology laboratories to the real world* [175,176] and exploiting the time-intensive and ecologically valid behavior data generated by digital systems [9-11]. The integration of fundamental psychological processes in the framework, such as reinforcement learning and sequential sampling models of decision-making, can increase the awareness of scientists in these fields to the practical value of their research and the great potential value of using digital systems in the field as data collection tools for *fundamental* social science research. We also hope that by summarizing and structuring the theoretical landscape for digital system designers, they can find their potential collaborations with behavioral scientists more efficiently. Finally, the clear mapping between intervention techniques and the processes and constructs in the framework makes it easier to search for the required data and manipulations to be used for theory testing.

### Scope and Limitation of the Framework

Given the ambitious goal of the framework to incorporate a wide range of theoretical traditions and to connect to the full gamut of digital intervention techniques, it is important to discuss the scope and limitations of our framework. First, the adaptive decision-making framework is not a new theory in itself or a model to be directly tested or falsified in a strong empirical sense. It is a framework that integrates existing theoretical ideas into a novel representation of lifestyle behaviors. In other words, it identifies relevant explananda in the course of lifestyle behavior change and provides explanations based on the most recent theoretical advances available. The usefulness of the framework should be judged by whether it succeeds in informing new computational models and intelligent intervention systems in the future, and predictions derived from the framework and its associated computational models should be rigorously tested using empirical data.

Second, proposing an integrated framework is not meant to discourage the use of individual theories in digital interventions. The adaptive decision-making framework is a framework of basic behavioral and cognitive processes in lifestyle behavior changes that are generalizable to a wide range of behavioral domains. However, there is also large heterogeneity across different behavioral domains and target populations, in terms of which processes in the framework are more critical and which variables or parameters are more changeable. For this reason, specialized theories are always needed, even if future theoretical advances might allow a single unified theory of basic psychological mechanisms. Therefore, it makes perfect sense for digital intervention systems to focus on one or a few processes or to target only a small set of variables for change based on domain-specific theories. The theoretical scope of the framework itself is focused on explaining individual lifestyle behaviors. It does not address interactions between individuals or larger socioeconomic processes.

Third, the adaptive decision-making framework is a theoretical framework of behavior change but not a framework of digital intervention systems. We have discussed the educational and heuristic value of the framework for digital intervention designers to help them understand and apply theories. It can potentially also motivate and facilitate the development of intelligent intervention systems that model user behaviors and cognitive states [161,174]. However, the framework should not be considered as a *cookbook* in the sense of prescribing specific design choices or requirements in specific interventions.

### Conclusions

We developed an adaptive decision-making framework in the hope that it will benefit behavior change theorists and digital system designers and, most importantly, facilitate better communication between the two communities. A stronger synergy will potentially help bring us closer to a future where digital systems live up to their potential to promote healthy lifestyles at scale. In the meantime, a wider adoption of more effective and theory-driven digital interventions will offer ample opportunities for building and testing new theories of human behavior.

## Abbreviations

BCT: behavior change technique
COMBI: computerized behavior intervention
JITAI: just-in-time adaptive intervention
SCT: Social Cognitive Theory
TPB: Theory of Planned Behavior
TTM: Transtheoretical Model
