# Correction: Theory Integration for Lifestyle Behavior Change in the Digital Age: An Adaptive Decision-Making Framework

**DOI:** 10.2196/29629

**Published:** 2021-04-15

**Authors:** Chao Zhang, Daniël Lakens, Wijnand A IJsselsteijn

**Affiliations:** 1 Human-Technology Interaction Group, Department of Industrial Engineering & Innovation Sciences, Eindhoven University of Technology Eindhoven Netherlands

In “Theory Integration for Lifestyle Behavior Change in the Digital Age: An Adaptive Decision-Making Framework” (J Med Internet Res 2021;23(4):e17127) the authors noted one error.

In the originally published manuscript, the heading “Mapping Digital Intervention Techniques to the Framework” was incorrectly set as a level 3 subheading rather than a level 2 heading. This has been changed to a level 2 heading in the corrected version of the manuscript.

The correction will appear in the online version of the paper on the JMIR Publications website on April 15, 2021, together with the publication of this correction notice. Because this was made after submission to PubMed, PubMed Central, and other full-text repositories, the corrected article has also been resubmitted to those repositories.

